# Four-month intrinsic viral cycle in COVID-19

**DOI:** 10.1016/j.xinn.2021.100196

**Published:** 2021-12-14

**Authors:** Ross N. Mitchell, Jinhai Zhang

**Affiliations:** 1 Institute of Geology and Geophysics, Chinese Academy of Sciences, Beijing 100029, China

## Introduction

Although “waves” of pandemics are common knowledge, a rigorous time-series analysis has not been conducted to test for cyclicity. The COVID-19 pandemic, caused by the transmission of the severe acute respiratory syndrome-coronavirus 2 (SARS-CoV-2), is one of the most significant events in modern human history. It is ongoing, and any means that can be used to understand its dynamics are critical for mitigating its harmful effects. We conducted a time-series analysis on COVID-19 case data, which relies on the efficacy of testing. Fourteen countries were analyzed as well as a global dataset including 189 countries. Standard time-series methods were employed.

## Trends and rhythms

**Figure 1:**
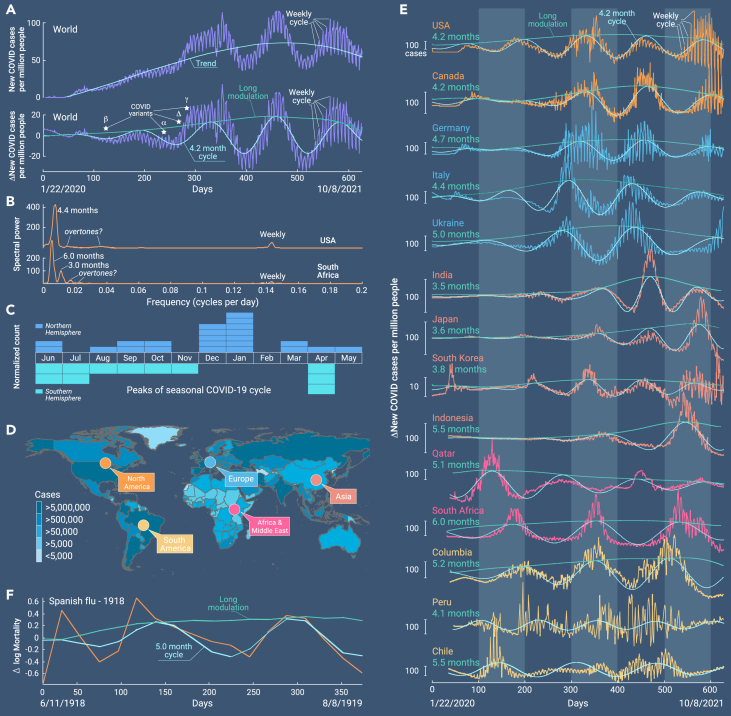
Time-series analysis of COVID-19 (A) Global trend in new COVID cases (top) and detrended time series with a 4-month cycle and long modulation (bottom). (B) Example periodograms from each hemisphere. Seasonal cycles were removed by notch-filtering to assess the spectral power of shorter wavelength cycles. (C) Histogram of peak COVID-19 cases of seasonal cycles by hemisphere. (D and E) The most prominent cycle in all 14 countries analyzed and the global dataset (189 countries) is a 4-month cycle with a long-term amplitude modulation. Map data in (D) from covid19.who.int. Data in (E) are color-coded by continent in (D). (F) Cycle and long modulation in mortality of the 1918 flu.

Datasets were detrended in preparation for spectral analysis. Countries exhibited one of two types of trends: linear or degree-2 polynomial regression. Countries with linear trends (e.g., USA) unfortunately have positive slopes, i.e., increasing cases. Only one exception, Qatar, has a negative linear slope. Countries with degree-2 polynomial trends exhibit either parabolic-down or parabolic-up trends. Japan and South Korea have parabolic-up trends, and Chile, Italy, and Peru have parabolic-down trends. The global dataset has a parabolic-down trend, implying that, globally, the peak intensity of the pandemic has been reached and its reduction has begun to “flatten the curve” (Figure 1A). With these first-order trends subtracted, we investigated the possibility of cyclicity in the second-order variations in COVID-19. Multiple cycles are present in the COVID-19 case datasets (Figure 1B). All 14 countries and the global dataset exhibit three bandwidths of cyclicity: (1) a high-frequency weekly cycle (that is amplitude-modulated by longer cycles and trends), (2) a middle-frequency cycle in the 3.5- to 6-month band (that is amplitude-modulated by a longest-wavelength cycle), and (3) a seasonal cycle ranging from 222 to 454 days.

The highest frequency cycle identified is 7 days, i.e., weekly (Figure 1B), where cases are lowest on Sunday and highest on Wednesday/Thursday. The weekly cycle can be both an effect and an artifact. On the one hand, increased quarantine on the weekend may create a real reduction in transmission, whereas during the workweek, cases rise as social distancing is more difficult to enact. On the other hand, much less virus testing and case reporting is done on weekends. While the identification of the weekly cycle is important for illustrating the validity of our methods, its interpretation carries little significance for the long cyclicity we report and is thus not considered further.

The lowest frequency cycle identified likely relates to seasonality, which is to be expected of respiratory viral infections. There has been research into whether COVID-19 exhibits seasonality and whether such climate-related changes are sufficient in reducing the pandemic during summer to prevent its recurrence in winter.^1^ In our analysis, all countries exhibit a pseudo-seasonal cycle, with periods ranging from 222 to 454 days. The seasonal cycles of the countries analyzed have an average of 314 days, and the global dataset has a 333-day seasonal cycle. The peak months in the seasonal cycle of each country are plotted by hemisphere in Figure 1C. Peak cases of the seasonal cycles are notably out of phase in the Northern and Southern Hemispheres, thus consistently indicating that peak cases occur in winter. Seasonal influenza, particularly in temperate climates with large seasonal changes, may peak in winter as the virus, transmitted through aerosolized droplets, is strongly correlated with humidity, whereas less humidity increases both viral transmission and survival.^2^ Another contributing factor is that the virus can take advantage of weakened immune systems of human hosts during cold winters. Two seasonal recurrences of the H1N1 influenza occurred during the three winters following the first year of three waves of the 1918 flu; although these recurrences were less severe, they prolonged the pandemic considerably.^3^

## Four-month intrinsic viral cycle

A mysterious middle-frequency bandwidth of cyclicity is identified in all 14 countries and the global dataset (Figures 1D and 1E). In the Northern Hemisphere, each country has one dominant mid-frequency cycle that ranges in period between countries from 3.5 to 5.1 months (Figures 1B and 1E) for an average of 4.3 months for the nine countries. The period for the global dataset is 4.2 months, whereas the global data and cycle most strongly resemble those of the USA, which has a larger percentage of cases than any country. In the Southern Hemisphere, countries exhibit a similar 4- to 6-month cycle but also a second mid-frequency cycle of lesser spectral power around ∼3 months (Figures 1B and 1E). Comparison of the spectra between hemispheres demonstrates how the Southern Hemisphere has a second, shorter period cycle of lesser spectral power whereas its counterpart in the north is much weaker (Figure 1B). The 3- to 6-month cycles in most countries exhibit very long-term amplitude modulations (Figure 1E). Amplitude modulation implies that the low- and mid-frequency cycles not only exist in the same system but that they are related to each other. The origin of this 4-month cycle is discussed later.

A natural next question to ask is whether other pandemics also exhibit the newly identified 3- to 6-month cycle. Unfortunately, datasets as complete as those of COVID-19 are not available. The 1918 flu (caused by an H1N1 influenza) is the most analogous to COVID-19 in terms of duration and lethality, although there are differences between the two pandemics. Antibiotics and influenza vaccines were not available at that time, and mitigation mainly relied on quarantine. The 1918 flu spread in three waves over a 1-year period,^3^ thus, 365 days ÷ 3 waves = 122 days ÷ 30.45 days/month = 4 months/wave, i.e., identical to the COVID-19 cycle. A rigorous time-series analysis of the mortality data yields strong spectral power with a period of 5.0 months (Figure 1F); a 2.7-month cycle (not shown) is also present but is of weaker spectral power. The combined bandwidth of the 2.7- to 5.0-month cyclicity thus centers on ∼4 months, similar to COVID-19. Furthermore, the amplitude of the three waves of the 1918 flu were also modulated by a longer cycle, with the second wave being an order of magnitude more lethal than the waves before and after it.^3^ Thus, the 1918 flu, with 4-month cyclicity and an associated long modulation, strikingly exhibited both prominent features of COVID-19, suggesting that the 3- to 6-month cycle and its long modulation may represent an intrinsic cyclicity of viral pandemics.

The identification of the 3- to 6-month cyclicity in viral pandemics is new. Factors in the spread of influenza include the virus, its hosts, virus-host interactions, environment, virus stability and transmissibility, and human interventions. There is no obvious human behavioral or environmental cycle to explain the origin of the 3- to 6-month cycle. It is too short for the seasonal cycle, which we identify in another, distinctly longer, bandwidth. The appearance of COVID-19 variants may be related to the 3- to 6-month cycle. The dates for the earliest samples identified for the four variants (α, β, γ, and Δ, so ordered for their designations as viruses of concern [VOCs]) show that they all originated during phases of the 3- to 6-month cycle when cases were low (Figure 1A), consistent with the notion that mutation occurs as an adaptive response to rising population immunity. There is thus a virus-host feedback between phases of the cycle with host immunity and viral mutation.

We suggest that the 4-month cycle is an intrinsic viral cycle due to virus-host feedback.^4^ Although this is speculative, we argue that it is quite reasonable. Perhaps the most compelling argument is that the cycle appears to operate independently of containment measures taken. These measures have notably varied significantly over time (from initial social distancing, masking, attention to personal hygiene, travel restrictions, and curfews to the later vaccines and other pharmaceutical measures) and between countries. The regular cyclicity observed in all 14 countries analyzed and the global dataset is thus hard to explain if such measures have any first-order control of the cyclicity. To be clear, such containment measures are likely critical for both keeping the *average* number of cases down and preventing fatalities, even though they appear to have little control over the 4-month cycle.

The systematic cycles identified here can be used to anticipate the natural fluctuations in COVID-19. The seasonal cycle suggests that in the coming winter months, cases will increase in the Northern Hemisphere. The long modulation of the 4-month cycle indicates that its volatility is generally reducing, possibly lending credence to the concept of herd immunity,^5^ at least as far as the amplitude of the 4-month cycle is concerned. Nonetheless, a word of caution should be mentioned as the trends of most countries analyzed have not yet started to “flatten the curve.” In light of the discovery of the 4-month cycle, government health officials may consider implementing policies of intermittent social distancing in phase with the particular period (3–6 months) of the intrinsic viral cycle for their country.

## Supplemental information

Data S1. COVID time series
